# A bleeding tumor of the foot

**DOI:** 10.11604/pamj.2018.31.11.16168

**Published:** 2018-09-04

**Authors:** Mohamed El Amraoui, Abdelhafid Achbouk

**Affiliations:** 1 Department of Dermatology, Mohammed V Military Training Hospital, Rabat, Morocco; 2 Department of Plastic Surgery, Mohammed V Military Training Hospital, Rabat, Morocco

**Keywords:** Botriomycoma, foot, bleeding tumor

## Image in medicine

Botriomycoma or pyogenic granuloma is a benign vascular tumor very common in daily practice, which often sits at the extremities and is favored by trauma. The evolution is often favorable enamelled by the frequency of recidivism; however some atypical or massively bleeding forms pose diagnostic and therapeutic difficulties. We present a case illustrating this problematic. Botriomycoma or pyogenic granuloma is a non-rare benign vascular tumor, favored by trauma, mainly at the extremities and characterized by post-traumatic bleeding recurrence. Spectacular and massively bleeding forms are rarely reported. We present a case. A 60-year-old woman, with a history of a recessive infra-centimeter nodule of the second left toe. Has consulted for an increase of this nodule up to three centimeters in diameter. She was lost to follow-up for four months, and then she reconsulted for the same tumefaction which became ulcerated and massively bleeding and having increased in volume to reach five centimeters of large diameter. Ultrasound showed a superficial and much vascularized tumor. In view of the very haemorrhagic character and the repercussions on the general condition of the patient, a total resection of the tumor was done urgently and the histology was in favor of a botriomycoma without signs of malignancy. The evolution was favorable. Most botriomyomas are small and at risk of recurrence, but rapidly progressive and massively bleeding tumor forms, as noted case, requires rapid management and collaboration between dermatologist and plastic surgeon.

**Figure 1:**
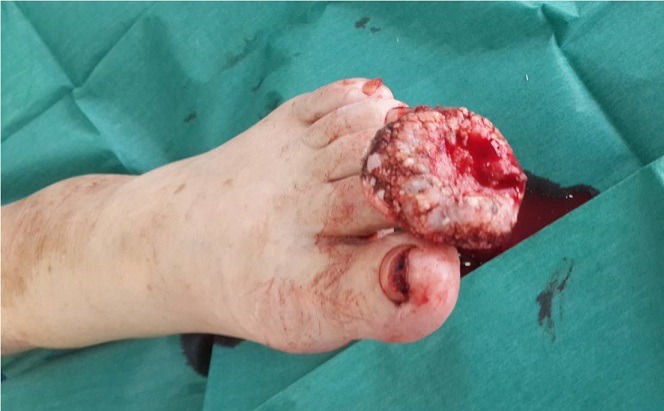
Bleeding tumor of the left second toe having rapidly increased in volume

